# Cords of the Brachial Plexus and Their Branches Positioned Laterally to the Axillary Artery

**DOI:** 10.7759/cureus.64795

**Published:** 2024-07-18

**Authors:** Vinay Sharma, CS Ramesh babu, Padamjeet Panchal

**Affiliations:** 1 Anatomy, Muzaffarnagar Medical College, Muzaffarnagar, IND; 2 Anatomy, AII India Institute of Medical Sciences, Patna, Patna, IND

**Keywords:** pectoralis minor, radial nerve, ulnar nerve, median nerve loop, axillary artery, intersegmental artery, developmental anomalies, variation in axilla and vascular variation, abnormal formation of the brachial plexus, brachial plexus block

## Abstract

The brachial plexus, which supplies the upper limb, extends from the interscalene triangle in the root of the neck to the axilla and is closely related to the subclavian and axillary arteries. Variations in the formation, branching pattern, and relations are profound, and it is generally stated that variant anatomy of the plexus appears to be a rule rather than an exception. In previous studies, it was hypothesized that the anomalous development of the subclavian-axillary stem and the persistence of intersegmental arteries could induce variations in the plexus. In this study, all three cords of the brachial plexus (lateral, medial, and posterior) and their terminal branches are consistently found lateral to the third part of the axillary artery. Most of the studies reported variation in one or the other cord or its branches, but very few studies have reported about all cords lateral to the brachial plexus. The brachial plexus variations are usually also associated with the variations in the branches of the axillary artery, but in this study, no such variation is noted in the branches of the axillary artery. These differences impact the methods of surgery and the application of regional anesthesia. For successful outcomes, it is important to know how neurovascular relationships work, such as where the cords are in relation to the axillary artery. We report an interesting case of all cords and their branches positioned lateral to the axillary artery in the axilla in an adult male cadaver.

## Introduction

The brachial plexus is a complex network of nerve fibers composed of the ventral rami of spinal nerves that emerge from the fifth cervical to the first thoracic spinal segments (C5-T1), preserving the dermatome and myotome patterns of the upper limb. The roots, trunks, and their divisions are located in relation to the subclavian artery at the root of the neck. By contrast, the cords and their terminal branches are located in the axilla, which is related to the axillary artery. These cords are strongly related to the axillary artery, and accordingly, they are named as per their relationship with the second part as lateral, medial, and posterior cords. Their terminal branches retained a similar relationship to the third part of the axillary artery. The variant anatomy of the brachial plexus is profound because of its complex formation, diverse branching pattern, and extensive anatomical distribution from the interscalene triangle at the root of the neck to the axilla. The varied anatomy of the brachial plexus is frequently observed and is more commonly the rule than the exception [1]. Miller hypothesized that subclavian-axillary stem developmental anomalies induce brachial plexus variations. Usually, the seventh intersegmental artery develops into a subclavian-axillary stem; in such cases, the axillary artery passes through the loop of the median nerve. Anomalous persistence of the sixth, eighth, and ninth intersegmental arteries resulted in variations in the formation and branching patterns of the brachial plexus. The author discovered all cords lateral to the axillary artery in one out of 408 dissections, providing evidence supporting this theory. The author proposed that the development of the subclavian artery from the ninth intersegmental artery, rather than the typical seventh intersegmental artery, was the cause of this unusual relationship [2].

Ultrasound-guided regional anesthetic approaches utilize the subclavian-axillary arteries as landmarks for locating the different parts of the brachial plexus, and their increased usage requires clinicians to possess thorough knowledge about neuro-vascular relations. Unfortunately, few studies have described variations in their relationship with the axillary artery. Yang et al. dissected 607 axillae of 306 cadavers and observed unusual axillary arteries that did not penetrate the loop of the median nerve in 12 cases, of which the cords were found lateral to the axillary artery in 11 cases. They suggested that this variant relationship was due to the development of the axillary artery from the ninth intersegmental artery [3]. Benes et al., in their meta-analysis of 40 studies (3055 upper limbs), noted the normal formation of roots and trunks in 84% of cases and divisions forming cords in 96% of cases. They also noted that only seven studies (746 upper limbs) reported a relationship between the cords and their branches and the axillary artery distal to the pectoralis minor, and the relationship was normal in 96% of cases. Only in three cases (<0.1%) were all three cords noted lateral to the axillary artery [4]. Although sporadically reported, the relationship between the brachial plexus and subclavian-axillary artery cannot be undermined, and successful surgeries and regional anesthesia can be achieved only by identifying the components of the plexus in relation to the arteries. Here, we present a case in which all cords and their branches lie laterally on the axillary artery.

## Case presentation

**Figure 1:**
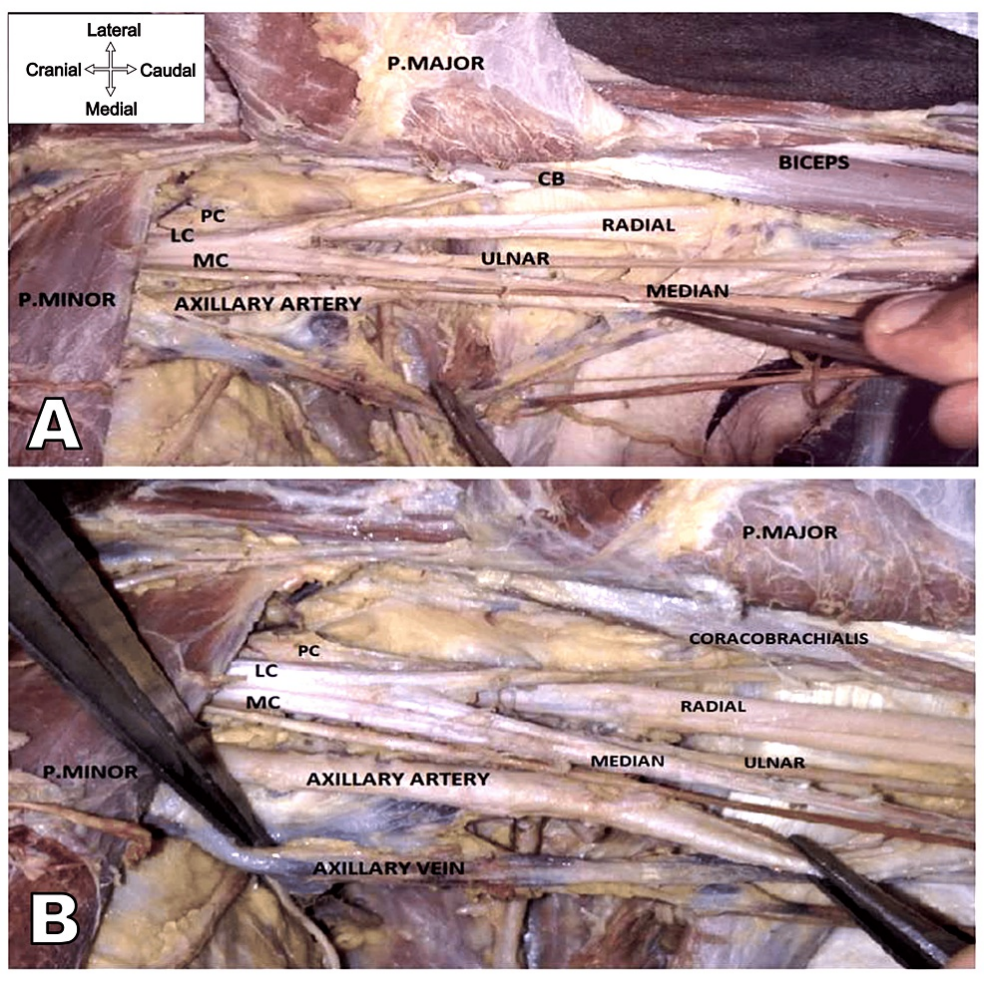
Dissection of left axilla showing the relationship of all three cords and their branches positioned lateral to the axillary artery. (A) The dissected view of the left axilla after reflection of the pectoralis major on the lateral side. (B) The dissected view of the left axilla, where the axillary artery and vein are separated from each other. Note that the axillary artery is not passing through the median nerve loop. LC: lateral cord; MC: medial cord; PC: posterior cord; P. major: pectoralis major; P. minor: pectoralis minor.

During the routine dissection of the left axilla for teaching undergraduate medical students, we observed the position of all three cords lateral to the axillary artery distal to the pectoralis minor in a North Indian male cadaver (Figure 1).

A similar relationship between the artery and cords was observed more proximally. No abnormalities were seen in the structure or branching of the three cords of the brachial plexus. They also maintained a consistent relationship with the axillary artery's first and second segments. The axillary artery did not traverse the median nerve loop. All three cords and their terminal branches were positioned lateral to the arteries. The radial and ulnar nerves were also lying lateral to the axillary artery, and the ulnar nerve was lying deep to the median nerve. The posterior cord is situated laterally and deeper than the medial, ulnar, and median nerves. The musculocutaneous nerve crossed the front of the radial nerve and moved laterally to enter the coracobrachialis. The axillary artery showed no signs of abnormal origin or branching patterns. The brachial plexus anatomy appeared to be within normal variation and consistent with typical anatomical structures. There was barely any communication of the musculocutaneous nerve with the median nerve. The subscapular artery, lateral thoracic artery, posterior humeral circumflex, and anterior humeral circumflex arteries were found to have a normal course. No communication was found between the median and ulnar branches surrounding the subscapular artery. The axillary vein coming from the arm to the axilla usually accompanies the axillary artery.

## Discussion

**Figure 2:**
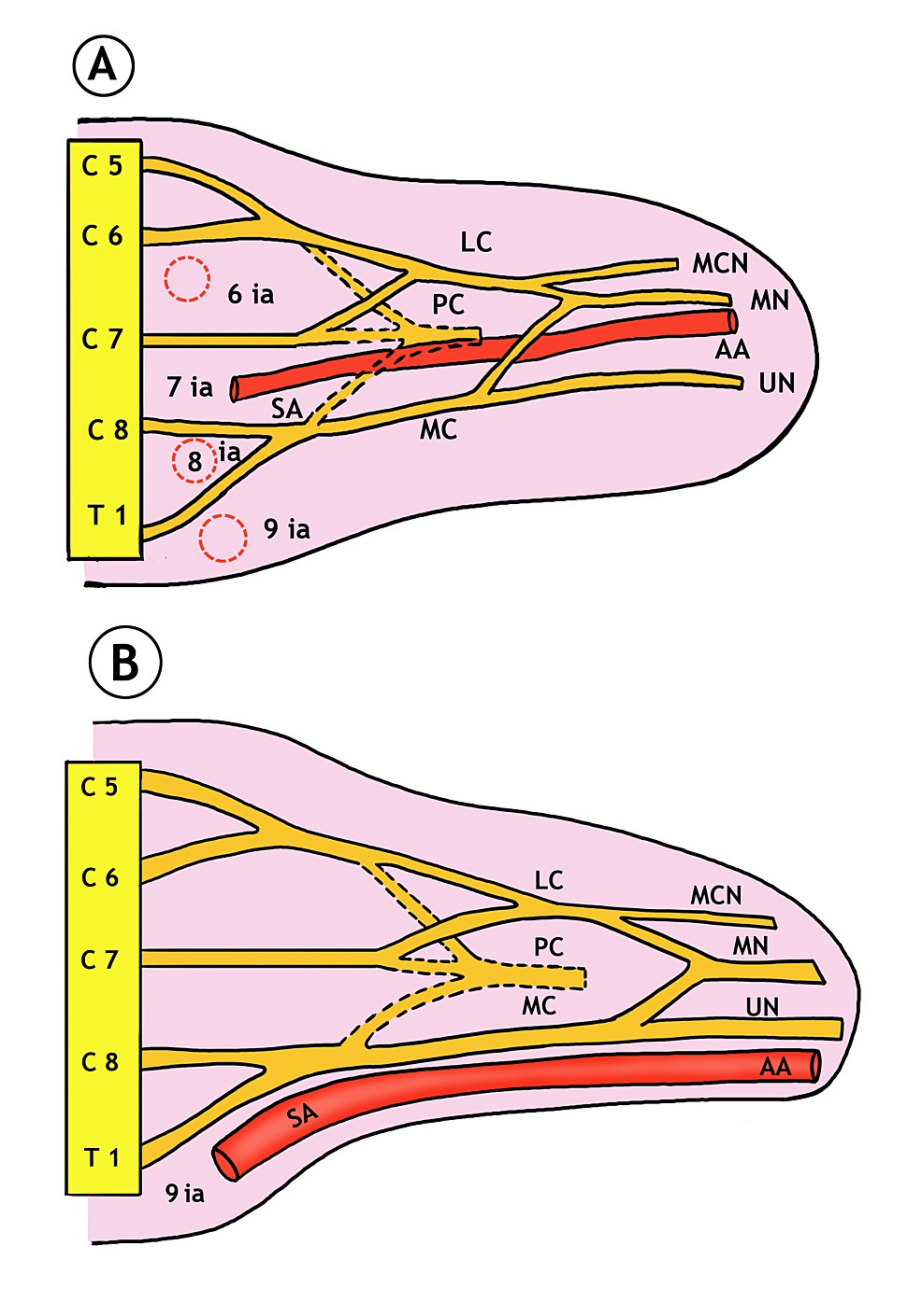
Schematic diagrams depicting the neurovascular relationship during development of upper limb bud. (A) Normal development where the seventh intersegmental artery (IA) develops into the subclavian-axillary stem and lies between the seventh cervical (C7) and eighth cervical (C8) roots and in a plane between the anterior and posterior divisions of the three trunks. When the subclavian-axillary stem grows further, it passes through the loop of the median nerve. Note the position of other intersegmental arteries between the roots of the brachial plexus, which later on disappear. (B) Persistence of the ninth intersegmental artery (IA), which lies caudal to the first thoracic (T1) ventral ramus, results in the subclavian-axillary stem, which does not pass through the median nerve loop. All cords and their branches are positioned lateral to the artery. Posterior divisions forming posterior cord are shown in dotted lines. IA: intersegmental artery; AA: axillary artery; SA: subclavian artery; LC: lateral cord; MC: medial cord; PC: posterior cord; MCN: musculocutaneous nerve; MN: median nerve; UN: ulnar nerve. This diagram illustrated by CS Ramesh Babu.

During development, the nerves forming the brachial plexus grow into a highly regulated environment guided by signaling molecules expressed by mesenchymal cells. The environment in which the brachial plexus grows contains constraints in the form of a pre-existing subclavian-axillary artery, the geometry and dimension of the upper limb bud, and the cartilaginous precursor of the arm skeleton [5]. In this proposed generic model, the developing subclavian-axillary stem is a critical determinant of plexus variation [5]. Miller suggested earlier that during development, the nerves and vessels have a reciprocal relationship, and variations in one influence variations in the other [2]. Usually, the seventh intersegmental artery grows as a subclavian-axillary stem and is positioned between C7 (the middle trunk) and C8 (the lower trunk with T1) and in a plane between the anterior and posterior divisions of the nerve bundles. When there is a confluence of the anterior divisions of the middle trunk (C7) and upper trunk (C5, C6), it results in the formation of a lateral cord, which contributes to the development of branches such as the lateral pectoral nerve, musculocutaneous nerve, and lateral root of the median nerve. The anterior division of the lower trunk (C8-T1) contributes to the formation of the medial root of the median nerve, which joins with the lateral root of the median nerve to form the median nerve. The seventh intersegmental artery, forming the normal axillary artery, appears to pass between the two roots of the median nerve (Figure 2).

Sometimes, the axillary artery develops from the sixth, eighth, and ninth intersegmental arteries, resulting in an anomalous relationship between the brachial plexus and the artery. The ninth intersegmental artery is positioned caudal to T1, and if it develops into an axillary artery, it lies medial to the plexus (in other words, the plexus lies lateral to the artery). Miller speculated that the brachial plexus associated with an axillary artery that develops from the ninth intersegmental artery remains compactly arranged, approximating and unifying the cords into a single entity [2].

In their study of 172 cadavers, Pandey and Shukla noted an anomalous position of all three cords lateral to the axillary artery in three male cadavers. They suggested that this positional anomaly could be due to the abnormal development of the axillary artery from an intersegmental artery other than the seventh [6]. Yang et al. dissected 607 axillae from 306 cadavers to elucidate the relationship between axillary artery development and accompanying brachial plexus variations. Of the 12 cadavers studied, they found that, in 11 cases, the plexus was located lateral to the axillary artery, suggesting abnormal neurovascular relationships. They hypothesized that the development of the axillary artery from the ninth intersegmental artery caused this anomaly. In five cases out of the 11, a single unified cord was found lateral to the axillary artery, supporting Miller’s hypothesis [2,3]. A similar variant relationship was also noted by Malukar and Rathwa in two out of 100 plexuses, Khan et al. in one out of 60 plexuses, and Jyoti and Sharma in one out of 30 limbs [7-9]. Prashant Moolya and Lakshmi Rajagopal dissected 114 upper limbs of 60 cadavers and observed the location of cords of the brachial plexus lateral to the axillary artery in four cases, of which two cases had a single unified cord [10]. The variant locations of all three cords lateral to the axillary artery were reported in case reports by Sathyanarayana et al., Jamuna, Gupta et al., and Eranga and Samarawickrama [11-14]. A considerable observation deduced from these case reports is that the variant relationship was present in three out of four cases on the left side. A single unified cord lying lateral to the axillary artery was also reported by Agarwal et al. in 4 out of 90 dissected limbs [15]. The bilateral presence of a single cord has also been reported [16].

Comprehensive knowledge of the normal and variant anatomical relationships between the subclavian-axillary stem and various brachial plexus components is crucial for the successful outcomes of regional anesthetic blocks, shoulder surgeries, oncological surgeries, vascular procedures, and neurosurgeries. Knowledge of variations in plexus formation and branching patterns is also essential for interpreting variant motor and sensory symptoms due to plexus injuries, which may otherwise lead to incoherent signs and symptoms and result in failure of patient management. Although few studies have focused on the interrelationship between the brachial plexus and the subclavian or axillary artery, no study has analyzed the relationship between the axillary vein. Leijnse et al. found a large vein penetrating through the lateral cord in nearly 54% of cases and draining into the axillary vein [5]. Future studies must include and analyze the relationship between the axillary and subclavian vessels and the brachial plexus.

## Conclusions

In this study, a unique anatomical variant of the brachial plexus was observed, where all three cords and their branches were consistently positioned laterally to the axillary artery in a North Indian male cadaver. This observation deviates from the typical anatomical arrangement, where the cords of the brachial plexus surround but are not uniformly lateral to the axillary artery. Such variations in the anatomical relationship between the brachial plexus and the axillary artery are considerable for clinicians performing surgeries, regional anesthesia, and managing trauma or pathology affecting the upper limb. Understanding these anatomical nuances is essential for successful outcomes in surgical interventions, particularly those involving the shoulder, arm, and hand. The study contributes valuable insights into the diversity of brachial plexus anatomy, highlighting the need for clinicians to be aware of such variations to avoid potential complications during procedures. Moreover, these observations underscore the complex interplay between developmental anatomy and clinical practice, emphasizing the importance of detailed anatomical knowledge in optimizing patient care in the medical field. Understanding how the axillary vein interfaces with the brachial plexus could provide valuable insights into the anatomical variations and clinical implications that affect vascular and neurological procedures in the upper limb.
